# Isolation and molecular characterization of *Mycoplasma* spp. in sheep and goats in Egypt

**DOI:** 10.14202/vetworld.2019.664-670

**Published:** 2019-05-13

**Authors:** Mounier M. Abdel Halium, Fayez A. Salib, S. A. Marouf, Emil S. Abdel Massieh

**Affiliations:** 1 Department of Medicine and Infectious Diseases, Faculty of Veterinary Medicine, Cairo University, Giza, Egypt; 2 Department of Microbiology and Mycology, Faculty of Veterinary Medicine, Cairo University, Giza, Egypt

**Keywords:** goats, *Mycoplasma*, polymerase chain reaction, sequencing and phylogenetic analysis, sheep

## Abstract

**Background and Aim:**

Different species of *Mycoplasma* are associated with many pathological problems in small ruminants including respiratory manifestation, this problem results in significant losses, especially in African countries. This study aimed to (I) study some epidemiological aspects of *Mycoplasma* species infections in Egyptian sheep and goats at Giza Governorate, (II) diagnosis of *Mycoplasma* species affections using bacterial isolation and identification, (III) apply the polymerase chain reaction (PCR) for typing of different *Mycoplasma* species, and (IV) illustrate the phylogenetic tree for the isolated *Mycoplasma* species and other species from GenBank using the purified PCR product.

**Materials and Methods:**

A total of 335 samples were collected from sheep and goats from Giza Governorate in Egypt as 142 nasal swabs from clinically affected animals, 167 pneumonic lungs, 18 samples from tracheal bifurcation, and 8 samples by bronchial wash were cultured on pleuropneumonia-like organisms (PPLOs) media for cultivation of *Mycoplasma* species. PCR and sequencing and phylogenetic analysis were adopted to identify and classify the isolated *Mycoplasma* species.

**Results:**

A total of 24 *Mycoplasma* isolates were isolated on PPLO media, identified by biochemical tests, and confirmed and typed by PCR using specific primers. 10 isolates were confirmed as *Mycoplasma arginini*, four isolates as *Mycoplasma ovipneumoniae* by PCR, and 10 isolates as undifferentiated *Mycoplasma species*. A purified isolate of *M. arginini* and *M. ovipneumoniae* was sequenced and phylogenetic analysis was illustrated.

**Conclusion:**

*M. arginini* and *M. ovipneumoniae* are prevalent in Egyptian sheep and goats. Further studies on *M. arginini* are required due to its high frequency of isolation from pneumonic sheep and goats and also from animals suffer from different respiratory manifestations.

## Introduction

*Mycoplasma* belongs to a group of bacteria named *Mollicutes* which characterized by its minute genome size and perpetually devoid of the cell wall [1]. Different *Mycoplasma* species are accompanying by many diseases and problems in both mammalian and avian species [2], sheep and goats (poor man’s cow) are economically critical in many countries including Egypt as consumed for meat, wool, and milk production, more than 10% of meat production in Egypt is from sheep and goats [3].

*Mycoplasma* infections leading to a great economic loss due to it cause high morbidity and mortality rates in sheep and goats populations in African countries including Egypt, European countries, and India [4]. *Mycoplasma* causes various clinical manifestations as pneumonia, conjunctivitis, arthritis, and mastitis [5]. *Mycoplasma* species commonly associated with pneumonia in small ruminants are *Mycoplasma ovipneumoniae*, *Mycoplasma arginini*, *Mycoplasma capri*, *Mycoplasma capripneumoniae*, and *Mycoplasma capricolum* [6]. *M. arginini* is frequently isolated with *M. ovipneumoniae* from cases of atypical pneumonia in sheep and goats [5] and other cases with lung consolidation [7]. *M. arginini* is also isolated from other locations such as genital organs, eyes, and ears [8]; in addition, isolation of *M. arginini* from pathological cases in human [9,10] gives suspicion that *M. arginini* may have zoonotic importance. In Egypt, different *Mycoplasma* species have been isolated including *M. arginini, M. ovipneumoniae*, and *Mycoplasma agalactiae* [11].

The respiratory problems are considered as multifactorial disease where interaction between different microbial agents such as bacteria (*Pasteurella* and *Mycoplasma*), viruses (PI3, reovirus, and adenovirus), and fungi and also other factors as host defense mechanism and environmental factors including climatic conditions and stress of transportations lead to increase the incidence of those problems [12].

*Mycoplasma* is highly fastidious microorganism required very precise media to develop *in vitro*, the low ability of *Mycoplasma* to form macromolecules needed for their growth refers to their evolutionary development from other bacteria; it is highly suspected that many *Mycoplasmas* exist in nature, however, have not been isolated due to their hard growth *in vitro* on artificial media [1].

Lately, the high development of polymerase chain reaction (PCR) technique makes the detection of different species of *Mycoplasma* using specific primers much simpler, till now, PCR remains the most valuable and rapid method for detecting specific species of *Mycoplasma* [13]. Moreover, the amplified purified PCR products of many genes from different strains can be sequenced, offering the chance for whole or partial typing of various species and strains at a more accurate level allowing molecular epidemiological studies and research.

The molecular epidemiological analysis allows the genotyping of different strains and has used in the tracing, control, and prevention of some diseases; also, molecular typing of some strains such as *M. arginini* would help in strain differentiation and then know the suspected pathological importance of these strains [8]. This study aimed to (I) study some epidemiological aspects of *Mycoplasma* species infections in Egyptian sheep and goats at Giza Governorate, (II) diagnosis of *Mycoplasma* species affections using bacterial isolation and identification, (III) apply the PCR for typing of different *Mycoplasma* species, and (IV) illustrate the phylogenetic tree for the isolated *Mycoplasma* species and other species from GenBank using the purified PCR product.

## Materials and Methods

### Ethical approval

Animal ethical approval was obtained from the Institutional Animal Care and Use Committee, Cairo University (II F 23 18).

### Animals and samples

A total of 246 sheep and 89 goats of different ages, sexes, and breeds suffering from respiratory manifestations were examined clinically and bacteriology for isolation and identification of *Mycoplasma* species.

The present investigation was carried out between December 2015 and November 2016.

A total of 142 nasal swabs (58 sheep and 84 goats) and eight bronchial washes (seven sheep and one goat) were collected from sheep and goats suffering from respiratory manifestations in different localities at Giza Governorate. A total of 167 lung tissues (165 sheep and two goats) and 18 tracheal bifurcations (16 sheep and two goats) were collected from slaughtered sheep and goats at Monieb abattoir.

### Clinical examination

Clinical signs of different respiratory manifestations in examined sheep and goats were recorded, a general examination of affected animals including body temperature was measured, cough test was applied, and chest auscultation was also adopted [14].

### Postmortem examination

Lung tissues, tracheas, and tracheal bifurcation of slaughtered sheep and goats were examined to detect and record different pathological changes [15].

### Epidemiological investigation

The prevalence of isolation of different *Mycoplasma* species in the examined sheep and goats was recorded according to species, ages, sexes, and season of the year [16].

### Bacterial isolation and identification of *Mycoplasma* species

For nasal swab from living sheep and goats with respiratory manifestation, sterile cotton swabs were used to collect nasal swabs from nostrils of affected sheep and goats, and they were taken on pleuropneumonia-like organisms (PPLOs) broth placed in an icebox and submitted directly to the laboratory. Serial dilutions from nasal swab were formed and incubated on PPLO broth of pH 7.4-7.6 with 15% heat-inactivated swine serum, 10% fresh yeast extract, and 0.0005 g/ml thallium acetate at 37°C for 3-7 days, then cultured on solid PPLO media by running drop technique [17].

For bronchial washes, approximately 1 cm long skin incision was done with a surgical scalpel over the midpoint of the trachea under local anesthesia of 2% lidocaine with epinephrine, a 16-gauge hypodermic needle was inserted into the tracheal lumen. A catheter equipped with two-way lumen connect valve was then inserted through the needle up to bronchial bifurcation; approximately 20-50 ml of Hank’s balanced salt solution was injected through the catheter and then immediately aspirated with syringe. The debris was then removed by low-speed centrifugation; and then, samples were cultured on PPLO broth for 3-7 days at 37°C and then cultured on solid PPLO by running drop technique [18,19].

For lung tissues and tracheal bifurcations from slaughtered sheep and goats, 167 samples from pneumonic areas and 18 samples from tracheal bifurcation were aseptically taken and placed in sterile plates kept in an icebox and were submitted to the laboratory. The outer surface of the pneumonic lungs was first seared with a heated spatula before cutting the inner surface of the lungs. Samples were taken either from bronchioles of each lobe of cut lung sections with micro tipped swabs or from lung homogenates by blending lung tissue with Hank’s balanced salt solution followed by removal of debris by low-speed centrifugation., Samples were then cultured on PPLO broth for 3-7 days at 37°C and then cultured on solid PPLO by running drop technique; filter syringe of 0.45 nm pore diameter was used for filtration of cultured broth media before culturing on solid media.

Mild turbidity in liquid media of cultured samples and fried egg appearance after examination of plates under the microscope indicates mullicates growth [20].

### PCR

For detection and typing of *Mycoplasma species* using PCR, positive cultures in liquid broth were centrifuged at 15,000 rpm for 10 min, and the pellet was resuspended in 300 µl of sterile distilled water, 300 µl TNES buffer (20 mM Tris, pH 8.0, 150 mM NaCl, 10 mM Tris-ethylenediamine tetra-acetic acid, and 0.2% sodium dodecyl sulfate) and proteinase K (200 µg/ml) were then added to the bacterial suspension and kept at 56°C for 1 h. The suspension was heated at 95°C for 10 min to inactivate proteinase K using a heat block method [20], the primer sequences, conditions of PCR, and the amplicon size with references are illustrated in Table-1 [2,5,10,20].

**Table-1:** PCR primers, conditions, and amplicon size.

Species	Primers	Annealing temperature	Amplicon size	References
Mycoplasma genus	GPO3F: 5’- TGGGGAGCAAACAGGATTAGATACC-3’ MGSO: 5’- TGCACCATCTGTCACTCTGTTAACCTC-3	At 53°C for 15 s	278	[20]
*M. arginini*	MAGF: 5’- GCA TGG AAT CGCATG ATT CCT-3’ GP4R: 5’- GGT GTT CTT CCTTAT ATC TAC GC-3’	46°C for 60 s	525	[10]
*M. ovipneumoniae*	MOVPF: 5’- GTT GGT GGC AAA AGTCACTAG-3’ MOVPR: 5’- CTT GACATC ACT GTT TCG CTG-3’	At 62°C for 90 s	418	[2]
*Mycoplasma capricolum* subspecies *capripneumoniae*	spe-F: 5’- ATCATTTTTAATCCCTTCAAG-3’ spe-R: 5’- TACTATGAGTAATTATAATATATGCAA-3’	47°C for 15 s	316	[20]
*Mycoplasma capricolum* subspecies *capricolum*	MCCPL1-L: 5’- AGACCCAAATAAGCCATCCA-3’ MCCPL1-R: 5’- CTTTCACCGCTTGTTGAATG-3’	At 47°C for 15 s	1350	[5]
*Mycoplasma mycoides* subspecies *Capri*	P4: 5’- ACTGAGCAATTCCTCTT-3’ p5: 5’- TTAATAAGTCTCTATATGAAT-3’	At 46°C for 90 s	195	[5]
*M. agalactiae*	Mag-F: 5’- CCTTTTAGATTGGGATAGCGGATG-3’ Mag-R: 5’- CCGTCAAGGTAGCGTCATTTCCTAC-3’	60°C for 60 s	360	[5]

PCR=Polymerase chain reaction, *M. arginine=Mycoplasma arginine, M. agalactiae=Mycoplasma agalactiae, Mycoplasma ovipneumoniae=M. ovipneumoniae*

### DNA sequencing and phylogenetic analysis

For PCR product purification and sequencing, QIAquick PCR Product Extraction Kit (Qiagen Inc., Valencia, CA) was used for purification of the PCR product directly while a purified PCR product was sequenced in the forward and/or reverse directions on an Applied Biosystems 3130 automated DNA Sequencer (ABI, 3130, USA), using already available BigDye Terminator V3.1 sequencing kit (Perkin-Elmer/Applied Biosystems, Foster City, CA) [21].

A BLAST^®^ analysis (Basic Local Alignment Search Tool) was initially performed to establish sequence identity to GenBank accessions. A comparative analysis of sequences was performed using the CLUSTAL W multiple sequence alignment program, version 1.83 of Meg Align module of Lasergene DNAStar software pairwise, which was designed by Thompson *et al*. [22] and phylogenetic analyses were done using maximum likelihood, neighbor-joining, and maximum parsimony in MEGA6 [23].

## Results

### Clinical examination

**Figure-1:**
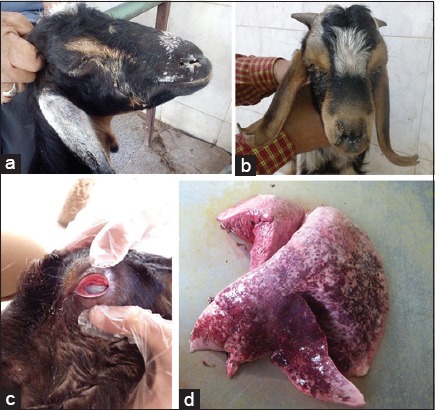
(a) Buck suffers from different respiratory manifestations with nasal and ocular discharges. (b) Goats suffer from severe respiratory distress with nasal and ocular discharge (corneal opacity). (c) Pneumonic sheep lung with areas of red hepatization, especially at the cranial lobe. (d) Colonies of *Mycoplasma* appear as a fried egg under the microscope.

Living animals were examined clinically; animals were suffered from different signs of respiratory manifestations including fever 40-42°C with depression, nasal and ocular discharges (mucoid, mucopurulent, and purulent), cough, and abnormal chest sound with auscultation (Figure-1a and b).

### Postmortem examination

The examined lungs, tracheas, and tracheal bifurcation showed different pathological lesions including congestion and edema with red and gray hepatization, hemorrhages in the trachea and tracheal bifurcation, and different degrees of pleurisy (Figure-1c).

### Some epidemiological data

The results of isolation of different *Mycoplasma* species in the examined sheep and goats in relation to sex, season, and age are illustrated in Tables-2 and 3.

**Table-2:** Number and percentage of positive samples with *Mycoplasma* species in examined sheep and goats in relation to sex and season.

Samples	Sex	Sheep								Goats								Total		
	
		Season								Season										
	
		Winter		Spring		Summer		Autumn		Winter		Spring		Summer		Autumn				
Nasal	Male	12	1	10	1	8	1	9	0	17	2	14	1	9	1	14	1	93	8	
swabs	Female	6	0	4	0	3	0	6	1	9	1	8	1	6	0	7	0	49	3	
Bronchial	Male	2	1	1	0	1	0	1	0	1	0	-	-	-	-	-	-	6	1	
wash	Female	1	0	-	-	-	-	1	0	-	-	-	-	-	-	-	-	2	0	
Tracheal	Male	4	1	3	0	2	0	3	0	1	1	-	-	-	-	-	-	13	2	
bifurcation	Female	1	0	1	0	-	-	2	1	-	-	-	-	-	-	1	0	5	1	
Lung	Male	31	2	28	2	21	1	23	1	1	0	-	-	-	-	-	-	104	6	
	Female	21	1	16	1	13	0	12	0	-	-	-	-	-	-	1	1	63	3	
Total		78	6	63	4	48	2	57	3	29	4	22	2	15	1	23	2	♂216	17	7.78%
																		♂119	7	5.88%
Percentage of positive samples		7.7		6.35		4.16		5.26		13.8		9.1		6.67		8.7				

**Table-3:** Distribution of *Mycoplasma* species in examined sheep and goats in relation to age.

Age	Sheep		Goats		Total	
			
	Number of samples	Number of positive samples	Number of samples	Number of positive samples	Number of positive samples	Percentage of positive samples
<15 months	102	7	29	4	11	8.37
From 15 months to 21 months	79	5	33	3	8	7.14
From 21 months to 27 months	34	2	14	1	3	6.25
>27 months	31	1	13	1	2	4.54

### Bacterial isolation and identification of *Mycoplasma* species

The collected samples were considered positive to *Mycoplasma* species when bacterial growth was observed, and it was indicated by turbidity in PPLO broth and fried egg appearance colonies in the agar plates (Figure-1d). Bacterial growth was observed in 24 samples (15 from sheep and nine from goats). Biochemically, all the 24 samples showed zones of growth inhibition around digitonin discs 5 mm in diameter or more, 14 were glucose fermentation test positive, and 10 samples were arginine hydrolysis positive.

### PCR

**Figure-2:**
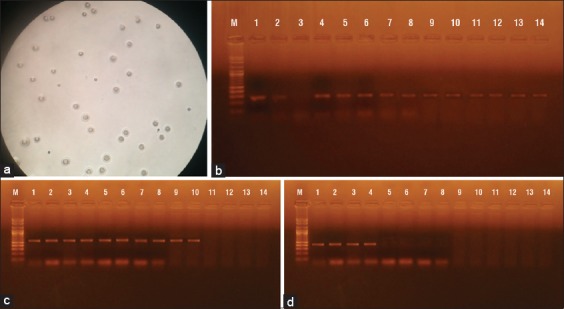
(a) Agarose gel of conventional polymerase chain reaction for detection of 16S gene using *Mycoplasma* genus-specific primer at amplicon size 278 bp. Lane M (Molecular weight marker, 100-1000 bp); Lanes 1-14, positive samples except for lane 3 negative control. (b) Agarose gel of conventional polymerase chain reaction for detection of *Mycoplasma arginini* species at amplicon size 525 bp. Lane M (Molecular weight marker, 100-1000 bp); Lanes 1-10 positive. (c) Agarose gel of conventional polymerase chain reaction for detection of *Mycoplasma ovipneumoniae* at amplicon size 418 bp. Lane M, (Molecular weight marker, 100-1000 bp); lanes 1-4 positive samples.

The total of 24 culture-positive isolates was confirmed as belonging to the *Mycoplasma* genus (Figure-2a) by the group-specific PCR, which produced specific bands with the molecular size of 278 bp.

In *M. arginini-*specific PCR amplification of the samples, six of the sheep isolates and four of the goat isolates produced positive products with the approximate molecular size of 525 bp (Figure-2b). The isolation percentages of *M. arginini* were, therefore, calculated as 40% (6/15) in sheep and 44.4 (4/9) in goats.

In *M. ovipneumoniae*-specific PCR amplification of the samples, three of the sheep isolates and one of the goat isolates produced positive products with the approximate molecular size of 418 bp (Figure-2c). The isolation percentages of *M. ovipneumoniae* in related to total *Mycoplasma* isolates were, therefore, calculated as 20% (3/15) in sheep and 11.11 (1/9) in goats.

The other 10 isolates which were positive with the general primer of *Mycoplasma* genus were tested against (*M. capricolum* subspecies *capripneumoniae*, *M. capricolum* subspecies *capricolum*, *Mycoplasma mycoides* subspecies *capri*, and *M. agalactiae*) using species-specific primers and were negative, the 10 samples considered as undifferentiated *Mycoplasma* species (Table-4).

**Table-4:** Typing of different *Mycoplasma* species using PCR.

Animal species	Type of sample	Number of samples	Number of *M. arginini* PCR-positive samples	Percentage of *M. arginini*-positive samples by PCR	Number of *M. ovipneumoniae* PCR-positive samples	Percentage of *M. ovipneumoniae*-positive samples by PCR	Number of undifferentiated Mycoplasma species	Percentage of undifferentiated Mycoplasma species
Sheep	Nasal swab	58	2	0.81	1	0.41	1	0.41
	Bronchial wash	7	0	0	1	0.41	0	0
	Tracheal bifurcation	16	1	0.41	0	0	1	0.41
	Lung	165	3	1.22	1	0.41	4	1.63
Total		246	6	2.44	3	1.22	6	2.44
Goats	Nasal swab	84	3	3.37	1	1.12	3	3.37
	Bronchial wash	1	0	0	0	0	0	0
	Tracheal bifurcation	2	0	0	0	0	1	1.12
	Lung	2	1	1.19	0	0	0	0
Total		89	4	4.5	1	1.12	4	4.45
Total		335	10	2.98	4	1.2	10	2.98

PCR=Polymerase chain reaction, *M. arginine*=*Mycoplasma arginine, M. ovipneumoniae=Mycoplasma ovipneumoniae*

The high-frequency rate of isolation of *M. arginini* from sheep and goats suffering from respiratory manifestations and pneumonic lungs makes us more interested, amplified purified PCR product of one isolate was subjected to sequencing analysis and the sequence then was submitted to NCBI GenBank taking accession number of MH685445 and also, purified PCR product of one isolate of *M. ovipneumoniae* was subjected to sequencing analysis and the sequence then submitted to NCBI GenBank taking accession number of MK045665.

### Phylogenetic analysis

**Figure-3:**
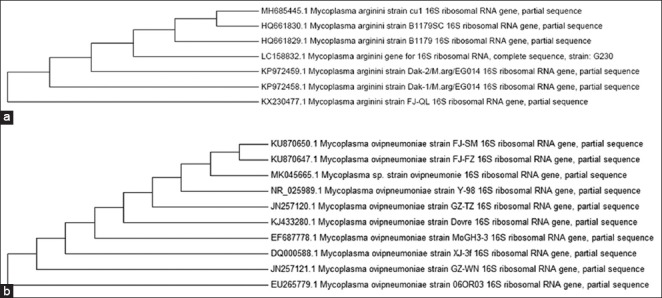
(a) Phylogenetic analysis of *Mycoplasma arginini* isolates based on 16S gene sequences, MH685445 isolate under study, KP972459 and KP972459 strains isolated from goats in Egypt, HQ661830 and HQ661829 strains from sheep in South Africa, LC158832 from sheep in Japan, and KX2304770 from goat in China. (b) Phylogenetic analysis of *M. ovipneumoniae* isolates based on 16S gene sequence, KU870650 and KU870647 strains isolated from goats in China, JN257120, EF687778, DQ0000588, and JN257121 from sheep in China, NR_025989 from wild sheep (*Ovis aries*) in the USA and EU265779 from bighorn sheep in the USA and KJ433280 from Norwegian Muskov in Norway.

A phylogenetic tree (Figure-3a and b) was formed based on 16S gene sequence of one purified strain of each species from the Egyptian isolates and other isolates of *M. arginini and M. ovipneumoniae* isolated from different countries and different species (from GenBank). Sequencing of 16S genes of *M. arginini* and *M*. *ovipneumoniae* Egyptian isolate and other isolates from other countries showed no significant difference between them.

## Discussion

*Mycoplasmas* of different species have been implicated in causing various diseases in sheep and goats including different respiratory manifestations, joint affection, conjunctivitis, and mastitis. The most common species isolated from small ruminants are *M. mycoides* cluster as *Mycoplasma capricolum* subspecies *capripneumoniae* the causative agent of contagious caprine pleuropneumonia, other species isolated from sheep and goats including *Mycoplasma ovipneumoniae* [24]; however, *M. arginini* has been isolated from a different pathological condition in small ruminants including pneumonia [17,25].

In this study, 335 samples including lung tissue, nasal swab, tracheal bifurcation, and bronchial wash were collected from sheep and goats in Egypt to isolation, identification, and classification of different *Mycoplasmas* species; *Mycoplasma* were isolated from 24 samples identified biochemically using digitonin, confirmed using PCR as *Mycoplasma* using *Mycoplasma* genus-specific primers.

The results obtained in this study showed that the prevalence rate of *Mycoplasm*a species was higher in goats than sheep. In Egypt, similar finding has been reported by Ammar *et al*. [11]; the occurrence of *Mycoplasma* was more frequently in young animals than adult; these findings are closely related to Elshafay *et al*. [26] who found higher percent of isolation in young animals than adult, especially in goats in Giza and Dakahlia Governorates. Analysis of obtained data on seasonal occurrence of *Mycoplasma* revealed higher occurrence in winter months (cold weather) than in summer months (hot weather) may be due to increasing stress factor at cold weather and grouping of animals and direct contact with each other. Ten samples were confirmed as *M. arginini* (2.98%) and four samples as *M. ovipneumoniae* (1.19%) by PCR using specific primer for each species, these results disagreed with Elshafay *et al*. [26] who recorded higher infection rates.

The high-frequency rate of isolation of *M. arginini* from different pathological cases in small ruminants, especially from pneumonic cases, and cases suffer from different respiratory manifestations [7,11,17,25] may refer to its pathogenic role in respiratory affections.

Experimental infection of goats <1-year aged by *M. arginini* isolates formed by Chinedu *et al*. [6] indicates that *M. arginini* have some pathogenic effect as all experimental animals suffered from cough and nasal discharges, on postmortem examination mild-to-severe congestion of lungs with lung edema. Histopathological abnormalities observed were acute interstitial pneumonia, hyperemia of pulmonary capillaries with sever infiltration with leucocytes in the interstitial tissues, and alveoli.

For estimation, the relationship between *M. arginini* and *M. ovipneumoniae* isolated from different species and different countries one purified isolate of each species from sheep pneumonic lungs was submitted for partial sequencing of 16S gene which showed > 99% sequence identity with different *M. arginini* and *M. ovipneumoniae* isolates from different species (sheep, goats, and wild ruminants) and different countries, phylogenetic analysis of 16S gene showed very high sequence identity between different strains regardless their geographical distribution.

## Conclusion

It was concluded that although the respiratory problem in small ruminant is very complex, as many factors play a role including infectious agents and predisposing factors, the high-frequency isolation of *M. arginini* from different respiratory manifestation and pneumonic lungs suspect its pathogenic role.

## Authors’ Contributions

MMA and FAS designed the experiment protocol and the study. ESA and SAM collected and analyzed the samples. All authors were involved in data analysis, scientific discussion, and writing of the manuscript. All authors read and approved the final manuscript.
